# Functional connectivity of the visual cortex in chronic migraine before and after medication withdrawal therapy

**DOI:** 10.1016/j.nicl.2023.103543

**Published:** 2023-11-17

**Authors:** Veronica Mäki-Marttunen, Dennis A. Kies, Judith A. Pijpers, Mark A. Louter, Nic J. van der Wee, Serge A.R.B. Rombouts, Sander Nieuwenhuis, Mark Kruit, Gisela M. Terwindt

**Affiliations:** a Cognitive Psychology Unit, Institute of Psychology, Leiden University, Leiden, The Netherlands; b Leiden Institute for Brain and Cognition (LIBC), Leiden University, Leiden, The Netherlands; c Department of Neurology, Leiden University Medical Center, Leiden, The Netherlands; d Department of Radiology, Leiden University Medical Center, Leiden, The Netherlands; e Department of Psychiatry, Leiden University Medical Center, Leiden, the Netherlands

**Keywords:** Migraine, Functional connectivity, Withdrawal therapy, Visual cortex, fMRI

## Abstract

Acute withdrawal of headache medication in chronic migraine patients with medication overuse may lead to a dramatic reduction in headache frequency and severity. However, the brain networks underlying chronic migraine and a favorable response to acute withdrawal are still poorly understood. The goal of the present study was to characterize the pattern of intrinsic magnetic resonance imaging (MRI) functional connectivity (FC) specific to chronic migraine and to identify changes in FC that characterize subjects with CM reverting to less frequent headaches. Subjects with chronic migraine (N = 99) underwent a resting-state functional MRI scan before and after three months of medication withdrawal therapy. In addition, we included four control groups who were scanned once: healthy participants (N = 27), patients with episodic migraine (N = 25), patients with chronic back pain (N = 22), and patients with clinical depression (N = 17). Using dual regression analysis, we compared whole-brain voxel-level functional connectivity with ten well-known resting-state networks between chronic migraine and control groups, and between responders to treatment (≥50 % reduction in monthly headache days) and non-responders (<50 % reduction), before and after treatment. Subjects with chronic migraine showed differences in FC with a number of RS-networks, most of which involved the visual cortex, compared with healthy controls. A comparison with patients with episodic migraine, chronic pain and depression showed differences in the same direction, suggesting that altered patterns of functional connectivity in chronic migraine patients could to some extent be explained by shared symptomatology with other pain, depression, or migraine conditions. A comparison between responders and non-responders indicated that effective withdrawal reduced FC with the visual cortex for responders. Interestingly, responders already differed in functional connectivity of the visual cortex at baseline compared with non-responders. Altogether, we show that chronic migraine and successful medication withdrawal therapy are linked to changes in the functional connectivity of the visual cortex. These neuroimaging findings provide new insights into the pathways underlying migraine chronification and its reversibility.

## Highlights

• First large-scale whole-brain fMRI study of FC in patients with chronic migraine.
• Comparison with healthy controls, episodic migraine, chronic pain and depression.
• Most of differences in FC in chronic migraine patients involved the visual cortex.
• Response to medication withdrawal showed differences in FC of visual cortex at baseline.

## 1 Introduction

In up to 25 % of patients suffering from episodic migraine, the attack frequency increases over time until headache is present on ≥15 days/month with ≥8 migraine days/month, and a diagnosis of chronic migraine (CM) is made (Ashina et al., 2021). Several risk factors for chronification of migraine have been established, such as comorbid depression and cutaneous allodynia (Louter et al., 2014, Louter et al., 2013). The most important risk factor is overuse of acute (headache) pain medication such as triptans analgesics (May and Schulte, 2016). Up to 70 % of CM patients fulfill criteria for medication overuse headache. However, it remains unclear whether medication overuse is cause or consequence of the process of migraine chronification and how exactly medication overuse influences chronification.

Patients with CM have a lower quality of life than episodic migraine patients, experience enormous impact of the disease on their daily functioning, and put a high strain on healthcare (Bigal et al., 2008, Munakata et al., 2009). Chronic migraine presents a therapeutic challenge, as many treatments that are successful in patients with episodic migraine appear of limited effect in chronic migraine, or can even lead to worsening of complaints as is implied by the role of medication overuse in chronification. Clinical experience and uncontrolled studies suggest that abrupt withdrawal of medication overuse in chronic migraine patients may lead to a dramatic reduction in headache frequency and severity after an initial withdrawal period of temporary worsening (Zeeberg et al., 2006, Dodick and Silberstein, 2008, Evers and Marziniak, 2010, Olesen, 2012, Chiang et al., 2016). However, markers associated with favorable response to withdrawal therapy are currently unknown. Furthermore, there is a gap in the knowledge on which mechanisms play a role in chronification and, most importantly, in the reversion to less frequent migraine.

The goal of the current study is to gain further insight into the neural signatures of CM and the effects of medication withdrawal. Resting-state functional MRI (fMRI) has been useful in elucidating the pathophysiology of a wide range of neurological and psychiatric disorders (Barkhof et al., 2014). As a first step we characterized differences in functional connectivity between patients with CM and medication overuse and a healthy population. Previous work on this topic has yielded inconclusive evidence (Schwedt et al., 2013, Chen et al., 2017, Androulakis et al., 2018, Coppola et al., 2019, Lee et al., 2019, Lerebours et al., 2019, Dai et al., 2021, Zou et al., 2021, Hu et al., 2023), possibly because of methodological limitations, such as small sample sizes, a focus on a limited number of brain areas or networks (differing across studies), or differences in choice of control groups. As a result of this lack of convergence between previous studies, the neural basis of CM is still poorly understood. In particular, it is unclear how much of the differences between CM patients and healthy controls can be attributed to symptoms such as pain or comorbid depressive symptoms. Chronic pain disorders and depressive symptoms are often comorbid conditions in migraine, and are associated to changes in brain connectivity (Pfannmöller and Lotze, 2019, Brakowski et al., 2017). Therefore, in the present study we compared whole-brain functional connectivity in CM patients not only with healthy controls but also with three additional control groups: patients with episodic migraine, depressive patients and chronic pain patients. This allowed us to differentiate intrinsic functional connectivity patterns associated specifically with chronification of migraine from those associated with depression and/or chronic pain and/or migraine in general.

Secondly, we investigated intrinsic whole-brain functional connectivity in CM patients with medication overuse headache before and after three months of medication withdrawal therapy. Previous research in patients with CM and medication overuse found that medication withdrawal increased BOLD activity associated with nociceptive stimuli in pain-related brain regions (Mehnert et al., 2018), and decreased regional gray matter volume (Mehnert et al., 2018), but only in responders (Riederer et al., 2013). We compared, for the first time, resting-state functional connectivity between patients that responded to medication withdrawal versus those who did not, before and after treatment. Responders were defined as those who showed a ≥50 % reduction in monthly headache days. Identifying the alterations in functional connectivity in CM, as well as the brain circuitry associated with response to treatment, may be key to our understanding of the disease mechanisms underlying CM and may eventually inform the process of drug discovery.

## 2 Materials and methods

This study was performed as part of the CHARM study (CHronification And Reversibility of Migraine study, https://trialsearch.who.int/, NTR3440) on the treatment of CM at the Leiden University Medical Center (Pijpers et al., 2019). The dates of enrollment were between December 2012 and February 2015.

### 2.1 Participants and clinical procedures

The study population consisted of Dutch Caucasian participants. In total, n = 112 participants aged 18–65 years, diagnosed with CM and medication overuse according to the International Classification of Headache disorders (ICHD-3) criteria, were included. Exclusion criteria were other major neurological disorders or other comorbidity, apart from mild to moderate depressive symptom, a history of illicit substance abuse, or overuse of non-triptan, non-analgesic acute headache medication. As described in more detail elsewhere (Pijpers et al., 2019), all CM participants started with a 4-week baseline assessment period, followed by a 12-week withdrawal period. The withdrawal therapy consisted of abrupt cessation of acute headache medication and tapering of prophylactic medication. As part of another project goal independent of the current study, immediately before initiation of the withdrawal treatment (t_0_), participants were randomly assigned (1:1) to receive botulinum toxin A (BTA, 31 injections; 155 units) or placebo injections (24 injections with saline plus seven injection with low-dose BTA to ensure blinding; 17.5 units). Withdrawal treatment effect was assessed after 12 weeks (t_3_), based on the headache characteristics in weeks 8–12. Response to treatment was defined as a ≥50 % reduction in monthly headache days (MHD) compared to the baseline assessment period. All CM participants underwent MRI before (t_0_) and after treatment (t_3_), regardless of the current migraine status. As published in our previous paper, BTA treatment had no significant additional effect on treatment response (Pijpers et al., 2019). Nonetheless, this factor was incorporated as a covariate of no interest in some of our statistical models (see below).

Healthy controls and episodic migraine patients were included via the Leiden Headache Center and database. Participants with episodic migraine (n = 25) were diagnosed according to the ICHD-3 criteria, and had a maximum of two migraine attacks per month, with a maximum of six monthly migraine days (MMD) and a maximum of ten MHD. Episodic migraine patients were excluded in case of another primary headache syndrome (apart from episodic tension-type headache on less than four days per month), other chronic pain conditions, and if they had a history of CM or medication overuse for headache. In total, 27 healthy controls were selected based on the absence of a primary headache syndrome (except from an occasional tension-type headache), chronic pain, and frequent use of pain medication and/or depression/anxiety (based on HADS scores). In addition, data were collected from patients with depression (n = 17) and patients with chronic pain (n = 22). Exclusion criteria for these two control groups were a primary headache syndrome according to ICHD‐II criteria (apart from occasional tension-type headache <10 days/month), any other condition associated with chronic pain, (suspected) neoplastic origin of the pain for the chronic pain participants, and intake of simple analgesics on >10 days/months. For all control groups, additional exclusion criteria were the abuse of recreational illicit drugs, use of psychotropic medication, and presence of any oncological or psychiatric disease, other than the specific types described in the inclusion criteria. All participants in the control groups underwent MRI imaging once (t_0_).

### 2.2 Ethics

The study was approved by the medical ethics committee of the Leiden University Medical Center. Written informed consent was obtained from all participants.

### 2.3 Data acquisition

All MRI scans were performed in a Philips Achieva 3T system and a 32-channel head coil. Anatomical data was acquired using a whole-brain 3D T1-weighted TFE sequence with a TR of 9.8 ms, TE of 4.6 ms, flip angle of 8°, 120 slices with a FOV of 224 × 182 × 144 mm and voxel size of 1.17 × 1.17 × 1.2 mm. Resting-state fMRI data were acquired using a single-shot EPI with a TR of 2340 ms, TE of 30 ms and flip angle of 80°. 38 slices were acquired with a FOV of 220 × 200 × 125 mm and using an isotropic voxel dimension of 2.75 mm with an interslice gap of 0.25 mm, resulting in a slice thickness of 3 mm. The resting-state scan lasted 6 min 58 sec and consisted of 175 volumes. For registration purposes, a high-resolution single-shot EPI fMRI image was acquired with a TE of 30 ms, TR of 5000 ms and flip angle of 80°. 84 slices were acquired with a FOV of 220 × 200 × 168 mm and a voxel size of 2 × 2 × 2 mm.

All scanning took place between 17:00 and 23:00 PM. Subjects were not allowed to take any acute headache medication or caffeine in the 4 h prior to scanning. Subjects were instructed to close their eyes and stay awake during data acquisition. The lights in the scanner room were switched off during acquisition of the resting-state data.

### 2.4 Preprocessing

All preprocessing analyses were performed using the FEAT toolbox of FSL version 5.0.7, following a standard pipeline consisting of the following steps: brain extraction; motion correction and automatic removal of motion artifacts using ICA-AROMA; and spatial smoothing using a kernel size of 5 mm full width at half maximum. To complete preprocessing, a high-pass temporal filter with a *σ* of 0.01 Hz was applied. Finally, resting-state images were registered to standard space using boundary-based registration: first, the functional images were registered to the high-resolution fMRI images (6 degrees of freedom); then the high-resolution images were coregistered to the T1 images; and finally the T1 images were non-linearly registered to MNI152 standard space, and the estimated parameters were applied to the functional images, which were resampled to 4 mm isotropic.

### 2.5 Dual regression analysis

We selected from the literature ten robustly reproducible resting-state networks with known functional correlates (Smith et al., 2012): the default-mode network (DMN), the central-executive network (CEN), the left and right fronto-parietal networks (FPN), the somato-motor network (SMN), the auditory network, three visual networks, and the cerebellar network. Two noise networks, one consisting of a white-matter template and one consisting of an extra-axial spaces template, were added to account for any residual white matter or cerebrospinal fluid noise. Using dual regression analysis, we estimated spatial maps representing voxel-to-network functional connectivity for each participant and session separately, following a two-step approach. In a first step, we used the ten selected resting-state networks in multiple spatial regression onto the individual dataset(s). This produced a time series for each network as expressed within the individual dataset. Then, in a second step, we used the participant-level time series as temporal regressors to produce spatial maps of regression coefficients for each network and each individual’s dataset(s). Thus, this two-stage regression approach resulted in a spatial map for each participant, resting-state network and session (applicable only for the CM patients), that indicated the degree of covariation between individual voxels throughout the entire brain and the resting-state network’s time series. Finally, we used these subject-specific spatial maps of voxel-to-network connectivity to study whether regional (voxel-wise) functional connectivity was different between CM patients and controls, and to study whether treatment-related changes in functional connectivity were different between CM responders and non-responders.

### 2.6 Statistical analysis

Whole-brain general linear model analyses were performed in SPM 12, including age and sex as covariates. Randomization arm (BTA or placebo) was added as an additional covariate of no interest in the whole-brain statistical comparisons between the responders to withdrawal therapy and non-responders. To compare functional connectivity in the CM patients and healthy controls at t_0_, we carried out one-tailed independent-samples *t*-tests on the subject-specific maps obtained from dual regression, with group as between-subjects factor. To examine the effect of medication withdrawal on functional connectivity, we fit a flexible factorial model with group (responders, non-responders) as between-subjects factor and session (t_0_, t_3_) as within-subjects factor. To examine differences between responders and non-responders before medication withdrawal, we carried out a two-tailed independent-samples *t*-test with group as between-subject factor. We report all clusters that survived correction for multiple comparisons using whole-brain family-wise error (FWE) correction with alpha set to 0.05. To examine the direction of the significant effects, we extracted individual data from the significant clusters using the rex toolbox (https://www.nitrc.org/projects/rex), and plotted the effects of interest.

### 2.7 Data availability

The data that support the findings of this study are available from the corresponding author, upon reasonable request.

## 3 Results

### 3.1 Final sample

Of the 112 CM patients that were initially included, two were excluded due to incidental findings on the initial scan, four dropped out after the first session, and seven were excluded due to technical issues or excessive motion in the structural or functional scans. Of the remaining 99 CM patients, complete and adequate datasets were obtained. The sample sizes of the groups based on response to medication withdrawal were n = 21 (responders) and n = 78 (non-responders). The characteristics of these groups are displayed in Table 1. Of the participants that were initially included in the four control groups, three healthy controls, one patient with episodic migraine, three patients with depression, and one patient with chronic pain were excluded due to technical issues or excessive motion. The characteristics of the final samples of the control groups are displayed in Table 2.

**Table 1:** Baseline demographic characteristics of the CM patients.

	Responders	Non-responders
Sample size	N = 21	N = 78
Age (mean, SD)	45.3 (10)	47.7 (11)
Gender M/F (%F)	5/16 (76 %)	18/60 (77 %)
MHD^a^ (mean, SD)	19.9 (4.4)	22.1 (4.6)
MMD^b^ (mean, SD)	15.2 (5.3)	15.7 (5.4)
Monthly days with use of acute anti-headache medication^c^^,^^d^	17.2 (5.1)	16.5 (5.6)
Migraine status (% of patients) (interictal/ictal/postictal/preictal/unknown)		
Session 1	66/22/5/0/7	33/22/11/8/26
Session 2	38/33/11/0/18	23/19/9/11/38
Disease duration (years)	30 (13)	30 (13)
Presence of aura (no/yes/unknown)	61/0/39	33/15/52

a Monthly headache days.

b Monthly migraine days.

c Simple analgesics (paracetamol, NSAIDs), triptans and/or combination drugs

d Hospital Anxiety and Depression Scale-Depression score.

**Table 2:** Demographic characteristics of the control groups.

	Chronic migraine	Healthy controls	Episodic migraine	Depression	Chronic pain
Sample size	N = 99	N = 22	N = 24	N = 14	N = 21
Mean age (SD)	47.2 (SD)^a^	41.1 (13.9)	43.3 (10.5)	35.5 (12.3)	47.5 (14.0)
Gender F/M (%F)	76/23 (77 %)^b^	13/9 (59 %)	21/3 (87 %)	6/8 (42 %)	15/6 (71 %)
Age range by F/M	20–65/19–63	22–60/19–62	25–60/30–60	24–65/26–61	24–48/19–58

Note: aChronic migraine patients and healthy controls did not significantly differ in age, t = 1.87, p = 0.06, and bgender, χ2 = 2.89, p = 0.09.

### 3.2 Functional connectivity in CM versus healthy controls

We assessed the patterns of resting-state functional connectivity between individual voxels across the brain and subject-specific maps of ten well-known resting state networks, and compared these functional connectivity patterns between CM patients and healthy controls. The MNI coordinates and peak *t*-statistics of all clusters showing differences between CM and healthy controls are summarized in Table 3.

**Table 3:** Differences in functional connectivity between chronic migraine (CM) patients and healthy controls (HC).

Network	Contrast	*p* value^a^ cluster	Size	*t* value	Coordinates	Location
Visual	CM-HC	0.026	111	4.83	44 −58 6	Middle Temporal Gyrus (R)
VisualMed	CM-HC	<0.001	264	5.26	28 48 18	Superior Frontal Gyrus (R)
ECN	CM-HC	0.001	187	4.93	−14 –76 12	Medial Occipitotemporal Gyrus (L)
	HC-CM	0.008	130	4.32	6 –92 −2	Medial Occipitotemporal Gyrus (R)
SMN	HC-CM	0.039	95	4.86	−40 –38 52	Inferior Parietal Lobule (L)
	HC-CM	<0.001	356	5.06	36 –38 60	Inferior Parietal Lobule (R)
DMN	HC-CM	0.001	201	4.86	0 –56 28	Posterior Cingulate (L)

a FWE-corrected.

**Fig. 1:**
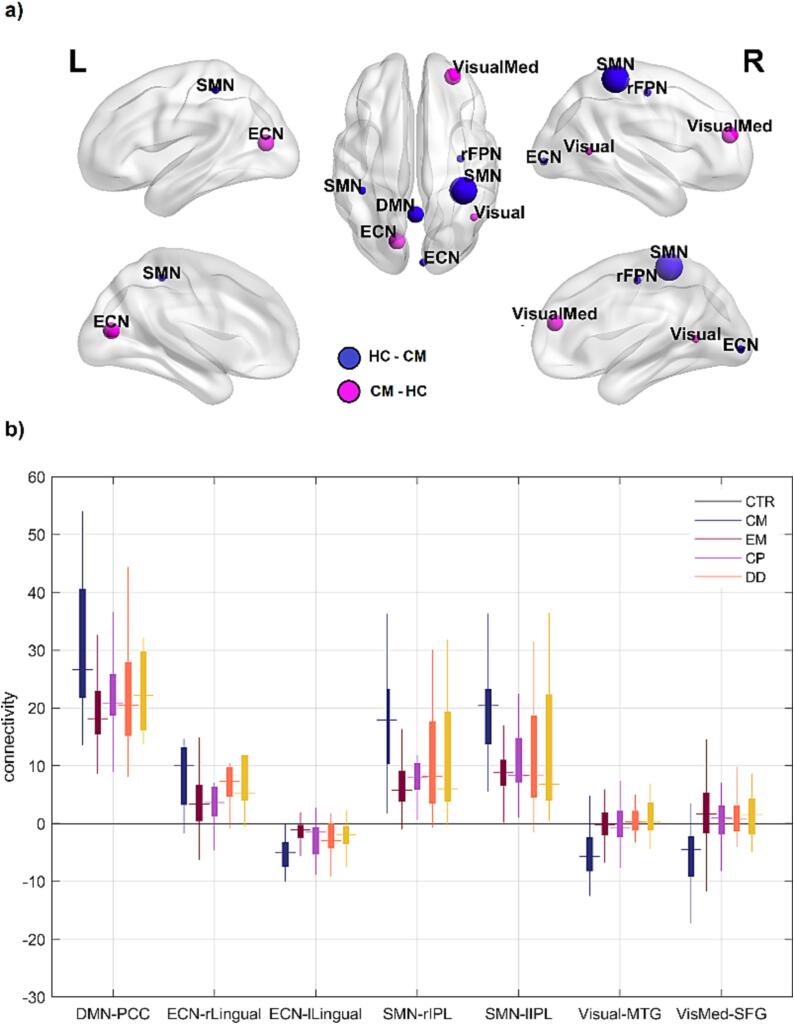
Connectivity differences between groups. a) Connectivity differences between patients with chronic migraine (CM) and healthy controls (HC). Spheres are centered in the regions where there was a significant effect (see Table 3). Labels indicate the network that showed significant connectivity effects with that region. Sizes of spheres relate to the size of the significant cluster. b) Average connectivity values for the different control groups. Connectivity values extracted from the clusters that were significant in the chronic migraine (CM) versus healthy controls (HC) contrast (Table 3). Error bars reflect standard error of the mean. CP: chronic pain, DD: depression disorder, DMN: default mode network, ECN: executive-control network, EM: episodic migraine, rFPN: right fronto-parietal, rIPL and lIPL: right and left inferior parietal lobule, rMedOTG and lMedOTG: right and left medial occipitotemporal gyrus, MTG: middle temporal gyrus, PCC: posterior cingulate cortex, SFG: superior frontal gyrus, SMN: sensorimotor network, VisMed: visual medial.

As shown in Fig. 1a, CM patients showed weaker (i.e., less positive or less negative) functional connectivity with a number of functional networks, relative to healthy controls. In CM patients, two visual networks showed less negative functional connectivity with clusters in the temporal and frontal lobes. The executive control network showed less negative connectivity with the left medial occipitotemporal gyrus (lingual gyrus) and less positive connectivity with the right medial occipitotemporal gyrus. The sensorimotor network showed less positive functional connectivity with bilateral clusters in the left and right inferior parietal lobule. Finally, the default mode network showed less positive connectivity with the posterior cingulate. Note that most of the connections showing a group effect (four out of the seven) involved the visual cortex.

To explore the specificity of the differences between CM patients and healthy controls, we calculated the strength of these seven functional connections for patients with episodic migraine, chronic pain and depression. The results show a remarkable pattern (Fig. 1b): for six out of seven connections, the three control groups show differences with respect to healthy controls in the same direction as the CM group. These findings suggest that the altered patterns of functional connectivity in the CM patients are to some extent similar to the patterns from the other patient groups, which may reflect the fact that these groups share common features related to pain, depressive symptoms, or migraine.

### 3.3 Functional connectivity changes after medication withdrawal in CM

**Fig. 2:**
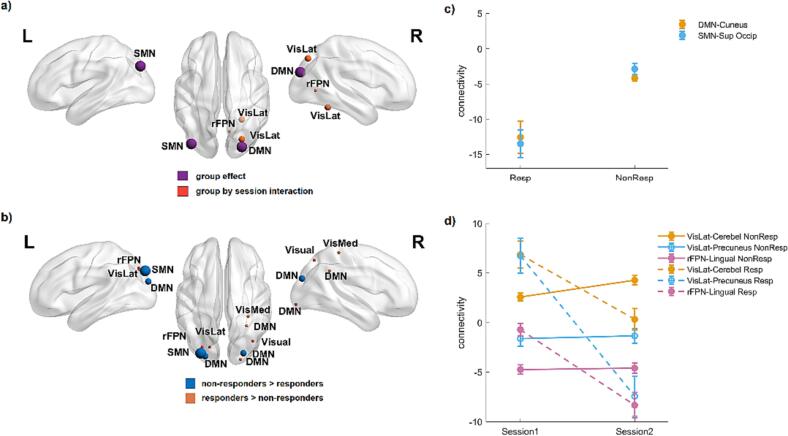
Brain effects of response to treatment on functional connectivity. a) Significant main effect of group (responders vs. non-responders) and the interaction effect between group and session (t_0_ and t_3_) on functional connectivity (see also Table 4). b) Connectivity differences between responders and non-responders at t_0_ (i.e., before medication withdrawal). Spheres are centered in the regions where there was a significant effect. Labels indicate the network that showed significant connectivity effects with that region. Sizes of spheres relate to the size of the significant cluster. c) Average connectivity values for each group (responders and non-responders), extracted from the clusters with a significant main effect of group (Table 4). d) Average connectivity values for each group and session, extracted from the clusters with a significant interaction effect. Continuous lines correspond to the non-responders and dashed lines to the responders. Cerebel: cerebellum, DMN: default-mode network, ECN: executive-control network, rFPN: right frontal parietal network, MedOTG: medial occipital temporal gyrus, SMN: sensori-motor network, Sup Occip: superior occipital gyrus, VisLat: lateral visual network.

Next we examined the effects on functional connectivity for responders versus non-responders. Table 4 lists the MNI coordinates and peak *t*-statistics of all connections that showed a main effect of group (responders vs. non-responders) or an interaction between group and session (t_0_ vs. t_3_). Interestingly, all five functional connections involved the visual cortex. As shown in Fig. 2a, two connections showed a main effect of group, with responders exhibiting a more negative functional connectivity between the default mode network and the cuneus in the occipital lobe, and between the sensorimotor network and the superior occipital gyrus (Fig. 2c). More importantly, three functional connections showed an interaction between group and session: two connections between the lateral visual network and the cerebellum and precuneus; and one connection between the right frontoparietal network and the medial occipitotemporal gyrus. All three interactions followed the same pattern: medication withdrawal resulted in less positive or more negative functional connectivity for the responders but not for the non-responders (Fig. 2d).

**Table 4:** Differences in functional connectivity as a function of session (t_0_ vs. t_3_) and group (responders vs. non-responders).

Network	Statistical term	*p* value^a^ cluster	Size	*t* value	Coordinates	Location
VisLat	group*session	0.002	170	5.46	26 –50 −20	Cerebellum (R)
	group*session	0.002	182	5.09	26 –76 44	Precuneus (R)
rFPN	group*session	0.003	171	5.11	10 –66 2	Medial Occipitotemporal Gyrus (R)
DMN	group	<0.001	244	5.16	26 –86 26	Cuneus (R)
SMN	group	0.001	217	4.60	−40 –82 34	Superior Occipital Gyrus (L)

a FWE-corrected.

We examined if these three functional connections (Fig. 2d) were also sensitive to three other potentially clinically relevant variables, above and beyond their sensitivity to response to treatment: disease duration (in years), HADS-D depression score at t_0_, and whether participants received BTA or placebo (at t_0_). Multivariate linear regression models with change in functional connectivity (t_3_ minus t_0_) as dependent variable, the above three variables and response to treatment as predictors, and age and sex as covariates, showed no significant effects of disease duration (p values between 0.12 and 0.43), HADs-D score (p values between 0.10 and 0.90) and BTA (p values between 0.88 and 0.97).

### 3.4 Functional connectivity differences between responders and non-responders at baseline

In a final analysis we compared functional connectivity between responders and non-responders before medication withdrawal (i.e., the simple main effect of group at t_0_). Significant connections with the ten resting-state networks are reported in Fig. 2b and Table 5. As in the previous analyses, most of the connections involved the visual cortex. When comparing responders to non-responders, the three visual networks showed higher functional connectivity with clusters in the parietal and occipital (cuneus) lobe. The default mode network of responders showed higher functional connectivity with clusters in the right lateral occipitotemporal gyrus (fusiform gyrus) and middle occipital gyrus, and lower connectivity with bilateral areas including the cuneus and middle occipital gyrus. Finally, the sensori-motor network also showed lower functional connectivity with the cuneus.

**Table 5:** Differences in functional connectivity between responders and non-responders before medication withdrawal (t^0^).

Network	Statistical term	*p* value^a^ cluster	Size	*t* value	Coordinates	Location
Visual	R-NR	0.009	120	4.56	38 –70 50	Superior Parietal Lobule (R)
VisualLat	R-NR	0.003	153	5.22	−18 –78 38	Cuneus (L)
VisualMed	R-NR	0.005	130	4.46	32 –38 60	Postcentral (R)
DMN	R-NR	0.028	95	4.78	22 –94 −8	Lateral Occipitotemporal Gyrus (R)
		0.02	102	4.95	30 –50 36	Middle Occipital Gyrus (R)
	NR-R	0	368	5.16	26 –86 26	Cuneus (R)
		0.001	177	4.69	−24 –90 22	Middle Occipital Gyrus (L)
SMN	NR-R	0	415	5.04	−30 –86 36	Cuneus (L)
rFPN	R-NR	0.016	115	4.87	−28 –78 40	Precuneus (L)

R: responders; NR: non-responders.

a FWE-corrected.

## 4 Discussion

Patients with CM and medication overuse displayed different resting-state functional connectivity than matched healthy controls—especially connectivity involving the visual cortex. We also found that CM patients who reverted to less frequent headache showed treatment-related changes in functional connectivity involving the visual cortex that were not present in non-responders. Finally, we found that responders and non-responders already differed in functional connectivity of the visual cortex before treatment, suggesting that changes in functional connectivity in visual areas may be used to distinguish CM patients who will benefit from treatment. Altogether, our results show a striking convergence, linking CM and successful treatment to functional connectivity involving the visual cortex.

Our findings provide new evidence regarding the important role of the visual cortex in migraine (Burke et al., 2020). Previous studies have reported hyperexcitability or hyperresponsivity of visual areas in response to transcranial magnetic stimulation (TMS) (Aurora et al., 1999) and light (De Tommaso et al., 2014, Mehnert et al., 2019), which may be a consequence of increased glutamate levels in visual areas (Zielman et al., 2017). Importantly, hyperexcitability as manifested in a decreased TMS-related phosphene threshold was found to be larger in CM than in episodic migraine (Aurora et al., 2005); and magnetoencephalography (MEG) correlates of visual cortex excitability were found to decrease in magnitude as CM patients reverted to episodic migraine (Chen et al., 2012). Future research is needed to establish whether the visual hyperresponsivity of the visual system in CM is associated with the here reported alterations in functional connectivity with the visual system, as well as with the treatment-related reductions in functional connectivity in responders.

Previous resting-state fMRI studies in CM patients have reported inconsistent evidence for altered functional connectivity of the visual cortex (Schwedt et al., 2013, Chen et al., 2017, Androulakis et al., 2018, Coppola et al., 2019, Lee et al., 2019, Lerebours et al., 2019, Dai et al., 2021, Zou et al., 2021). However, this is probably due to methodological limitations. All but one study (Chen et al., 2017) had small to moderate sample sizes (between 10 and 30 CM patients). Furthermore, the majority of previous studies focused on a small number of brain areas or resting-state networks, often not including the visual cortex, limiting the possibility to yield converging evidence across studies. Only two previous studies examined whole-brain functional connectivity (Lee et al., 2019, Zou et al., 2021), of which one used episodic migraine patients instead of healthy individuals as a control group. Large-scale whole-brain follow-up studies are needed to establish the reproducibility of the current findings, a concern that has also plagued studies on functional connectivity in episodic migraine patients, which have reported a wide diversity in brain areas showing altered connectivity (Mehnert et al., 2019, Skorobogatykh et al., 2019). Nonetheless, the results of our large sample of CM patients consistently support an involvement of visual cortex in CM, suggesting that connectivity involving this region is a key signature of CM.

We anticipated that some functional connectivity differences associated with CM may be related to symptoms that are shared between migraine and other neuropsychiatric or pain conditions, such as depression and chronic pain. To assess this possibility, we included three other control groups: episodic migraine, chronic pain and depression. We found that, overall, functional connectivity differences involving the default mode network, sensorimotor and visual networks were similar for CM and the other control groups as compared to healthy controls. In line with these findings, previous studies have found altered connectivity within the default mode network in major depression (Mulders et al., 2015), and a reorganization of connectivity in the sensorimotor cortex of patients with chronic pain (Mano et al., 2018). This calls for caution when interpreting differences in brain connectivity between CM and healthy controls.

Our results consistently point to effects of withdrawal therapy on visual cortex connectivity. Differences of connectivity of the visual cortex were also significant when comparing CM to healthy controls, thus suggesting that the therapeutic effects of withdrawal from pain medicine affect the regions implied in chronic migraine itself. The circuits involved in effectiveness of withdrawal therapy are unknown, but mostly subcortical systems have been implicated (Mehnert et al., 2018). Our results suggest that further studies investigating the effect of medication withdrawal on subcortical pain systems should also contemplate the involvement of cortical areas to understand the mechanisms of CM (Puledda et al., 2019).

We found that patients that responded to treatment showed a reduction in connectivity involving visual areas. We speculate that the difference between sessions in responders suggests that the hyperconnectivity in the visual cortex may have been part of the pathophysiology of CM in those responsive patients, sustained by medication overuse, and that after medication withdrawal, the normalization of that connectivity corresponded to the reversion of symptoms. The pathophysiology of non-responders, which was the majority of CM sample, may involve some other mechanism, independent of the medication. Interestingly, a similar proportion of males and females in the responders/non-responders groups indicates that the effects may not relate to the mechanisms implicated in the difference of incidence of CM across sexes. Taken together, our results are in agreement with heterogeneity within CM (Messina et al., 2023, Andreou and Edvinsson, 2019) and suggest that characterizing patient subtypes may help in patient-specific treatment.

The strengths of our study include the large number of CM patients (N = 99) compared to previous functional connectivity studies, the comprehensive whole-brain approach, and the inclusion of multiple control groups. It is also the first study in migraine patients that examined the effects of medication withdrawal on functional connectivity patterns in the brain; the experimental and longitudinal nature of the design allows causal inferences about the effects of withdrawal. However, we also acknowledge several limitations. First, our control groups had relatively small sample sizes (ranging from 14 to 24). Second, it is difficult to draw specific conclusions about neuronal mechanisms based on the direction (i.e., more/less negative, more/less positive) of changes in resting-state functional connectivity. Third, we found robust group differences between responders and non-responders at baseline and after three months of withdrawal therapy, and future studies should show how our findings generalize to novel individuals and have clinical (e.g., predictive) utility (Scheinost et al., 2019). Fourth, we did not collect information from the four control groups regarding the presence of pain during scanning. This potentially limits the comparison with the CM patients, of which 22 % (session 1) and 33 % (session 2) were in the ictal phase during scanning. However, presence of pain was never severe in the CM group, and therefore it is unlikely that it affected our main results. And fifth, the cross-sectional nature of our comparisons between CM and control groups means that we cannot determine if the differences in functional connectivity are consequences or causes of (a predisposition to) CM.

Our results suggest several promising avenues for future research. First, our results show a striking convergence, linking CM and successful treatment to functional connectivity involving the visual cortex. This suggests that the visual cortex may be a therapeutic target for neuromodulation in CM. Interestingly, the back of the head (occiput) is the target of single-pulse TMS with the FDA-approved eNeura device for treating migraine (Lipton and Silberstein, 2015, Starling et al., 2018). The possibility that medication withdrawal therapy targets some of the same posterior brain areas (and their connections with other parts of the brain) as those targeted by eNeura could be tested in future research by comparing functional connectivity before and after treatment with eNeura. Second, it would be interesting to examine if the functional connections that we have identified with resting-state fMRI in relation to CM and medication withdrawal, will also stand out during task-related fMRI (Cole et al., 2014). Third, future research could use Granger causality analysis to investigate the direction of information flow between each pair of functional connectivity nodes. Fourth, future research should examine the physiological (e.g., hormonal or neuromodulatory) processes that could underlie the overlapping functional connectivity changes in these clinical conditions. And finally, future studies on CM could explore the relationships between functional connectivity of the visual cortex and quantitative measures of the responsivity of these visual areas to TMS and light.

## 5 Competing interests

G.M. Terwindt reports consultancy support from Abbvie/Allergan, Lilly, Lundbeck, Novartis, and Teva, and independent support from Dutch Organization for Scientific Research, the Dutch Heart & Brain Foundations, International Retinal Research Foundation (IRRF) and Dioraphte; D. Kies and J. Pijpers, report independent support from the Netherlands Organisation for Health Research and Development and the Dutch Brain Foundation; V. Mäki-Marttunen, S. Nieuwenhuis, M. Kruit and M. Louter report no competing interests; N.J.A. van der Wee reports consultancy for Pfizer, Eli Lilly, Servier, Weyth, and independent support from the Netherlands Organisation for Scientific Research and the EU Horizon 2022 program; S.A.R.B. Rombouts reports independent support from the Netherlands Organisation for Scientific Research.

## Funding

Supported by grants from the Netherlands Organization for Scientific Research (Terwindt VIDI 917-11-31; Mäki-Marttunen and Nieuwenhuis VI.C.181.032) and the Dutch Brain Foundation (2013(1)-247).

## CRediT authorship contribution statement

**Veronica Mäki-Marttunen:** Formal analysis, Visualization, Writing – original draft, Writing – review & editing. **Dennis A. Kies:** Data curation, Methodology, Writing – review & editing. **Judith A. Pijpers:** Data curation, Writing – review & editing. **Mark A. Louter:** Data curation, Writing – review & editing. **Nic J. van der Wee:** Data curation, Writing – review & editing. **Serge A.R.B. Rombouts:** Methodology, Writing – review & editing. **Sander Nieuwenhuis:** Writing – original draft, Writing – review & editing. **Mark Kruit:** Data curation, Writing – review & editing. **Gisela M. Terwindt:** Conceptualization, Funding acquisition, Writing – review & editing.

## Declaration of Competing Interest

The authors declare that they have no known competing financial interests or personal relationships that could have appeared to influence the work reported in this paper.

## Data Availability

Data will be made available on request.
